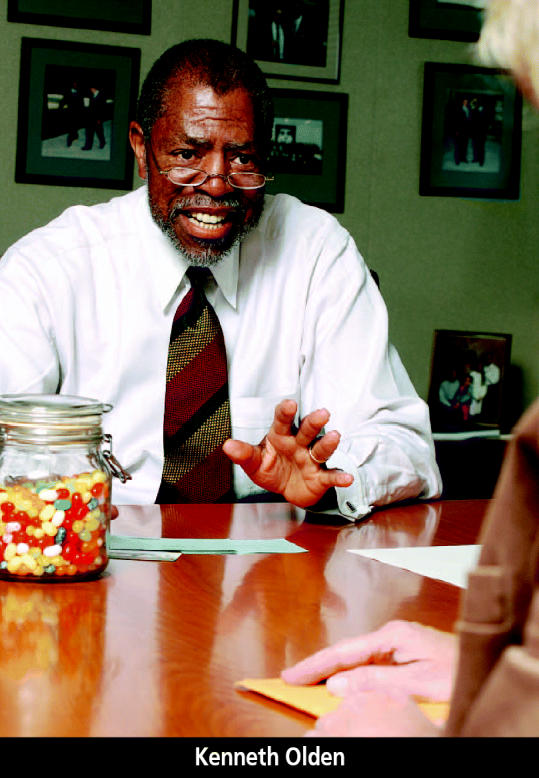# Editorial: Olden Times: Looking Back on a Career at the NIEHS

**DOI:** 10.1289/ehp.112-1247486

**Published:** 2004-08

**Authors:** David Brown, Kimberly G. Thigpen Tart, Thomas J. Goehl

**Affiliations:** NIEHS, National Institutes of Health, Department of Health and Human Services, Research Triangle Park, North Carolina, E-mail: brown4@niehs.nih.gov; NIEHS, National Institutes of Health, Department of Health and Human Services, Research Triangle Park, North Carolina, E-mail: thigpenk@niehs.nih.gov; NIEHS, National Institutes of Health, Department of Health and Human Services, Research Triangle Park, North Carolina, E-mail: goehl@niehs.nih.gov

After 13 years of distinguished service as director of the NIEHS and the National Toxicology Program (NTP), Kenneth Olden is stepping down to return to the laboratory to continue his work on cancer biology. His tenure at the NIEHS has been marked by numerous scientific advances, as well as by the creation of inspired programs to translate these advances into practicable means for real improvement in the health and lives of human beings around the world. Intrinsic to this process has been his belief in the necessity of making often complicated and technical science understandable to all so that it might become a tool by which people can make informed decisions. His contribution to the development of *EHP* from a series of monographs to one of the leading international science journals is but one example of the concrete application of this philosophy.

Olden was appointed director of the NIEHS and NTP in June of 1991, becoming the first African American to head an NIH institute. He made it his challenge to introduce an orderly, reasoned approach to the management of human health research related to the environment. He worked to change a culture of conflict and opposition among environmental health stakeholders to one of trust, respect, and cooperation. Olden broadened the definition of “environment” to include not just chemical and physical agents, but also food and nutrients, biological agents, prescription drugs, lifestyle choices, social and economic factors, and the built environment**.**

Along with this broadened definition, he established a new paradigm for environmental health research. He strengthened peer review, increased public input, and orchestrated a major reorganization of the institute’s intramural research program. During his tenure, the NIEHS has become the focus of national and international attention for outstanding research accomplishments including the discovery of a breast cancer gene (*BRCA1*) and the isolation of a metastasis gene in prostate cancer. At the same time, Olden posted a new list of priorities that went beyond an emphasis on identifying environmental agents that cause cancer—the new list included identifying disease end points such as reproductive and developmental defects, developing molecular prevention and intervention programs, and establishing clinical programs in environmental health.

Many of Olden’s research initiatives grew out of an observation that became his mantra: that human diseases are the product of a triangle of influences comprising environment, genetics, and aging. Thus, under his leadership the NIEHS initiated the Environmental Genome Project (EGP) in 1997. This project is a major national effort to identify those genes that confer susceptibility to various diseases as a consequence of exposure to specific environmental agents. The first phase of this project identifed more than 20,000 single-nucleotide polymorphisms (SNPs) in 217 environmentally relevant candidate genes; the goal is to resequence and analyze for SNPs in a total of 554 genes. A companion program, the Comparative Mouse Genomics Centers Consortium, was initiated by the EGP to develop transgenic and knockout mouse models based on human DNA sequence variants in environmentally responsive genes. These mouse models are tools to improve our understanding of the biological significance of human DNA polymorphism.

Extending the work of the EGP, Olden established the National Center for Toxicogenomics (NCT) in 2000. Through this center, NIEHS researchers and their partners at various government and academic institutions in the Toxicogenomics Research Consortium are surveying the human genome for alterations in gene expression patterns of several thousands of genes using DNA microarray technology. The advantages of the toxicogenomics approach over traditional toxicity tests are greater speed and efficiency, and a reduction in animal use. The NCT is already spurring the development of gene-based toxicity studies. Initiatives to develop other “omics” technologies such as proteomics and metabolomics were begun under Olden’s leadership and are coming to fruition in both intramural and extramural research programs, and through the NTP. Olden’s vision to form multidisciplinary collaborations among numerous research entities will no doubt revolutionize the way research in toxicology and environmental health is conducted in the future.

Realizing that the research needs to be done but that it also must be effectively communicated to the scientific community and other stakeholders, Olden took his vision for the advancement of toxicogenomics a step further and directed the development of an *EHP* toxicogenomics section. Published in quarterly issues, this section is designed to facilitate scientific discourse in these rapidly emerging fields so that researchers may take advantage of each others’ efforts and pool their knowledge for a more complete understanding of genomics and systems biology.

Seeing the larger picture and understanding that the best results come from the combination of many and varied talents have been a hallmark of Olden’s tenure at the NIEHS. Olden created centers within the NIEHS and the NTP consisting of leaders in toxicology research and risk assessment from government, industry, academia, and the private sector. Examples include the Interagency Center for the Evaluation of Alternative Toxicological Methods, the Center for the Evaluation of Risks to Human Reproduction, and the Center for Phototoxicology. These centers provide objective interpretation of scientific data to be used in conducting more credible scientific health assessments, and in promoting the development of new methodologies to better evaluate environmental health issues. Because the centers focus only on scientific issues and not on regulatory policies, they are looked upon as a neutral forum.

In the grants programs, the Superfund Basic Research Program and the Worker Education and Training Program have experienced substantial growth over the last 10 years, and their budgets are now appropriated directly from Congress. Olden also expanded on the extensive network of NIEHS-funded Environmental Health Sciences Centers and established a number of specialty centers including the Centers for Children’s Environmental Health and Disease Prevention Research (cofunded with the U.S. Environmental Protection Agency), the Collaborative Centers for Parkinson’s Disease Environmental Research, and the Breast Cancer and the Environment Research Centers. All of the centers are based on a model of multidisciplinary research collaboration and coordination that has recently been recognized by the NIH in its Roadmap for Medical Research as the wave of the future in biomedical studies.

Think like a wise man but communicate in the language of the people.

William Butler Yeats (1865–1939)

Since taking the helm of the NIEHS, Olden has been at the forefront in another area as well. From early on he showed an awareness and understanding of a fact that had often been ignored by others in research administration—that local communities have the collective ability to identify environmental health problems but often lack the time, means, and research expertise to effectively resolve these problems. He immediately put into place a series of measures, expanded over the course of his tenure, that would link community groups with resources at the NIEHS and other research institutions.

Olden made it mandatory for each NIEHS-funded Environmental Health Sciences Center to have a Community Outreach and Education Program that was responsive to local environmental health problems, particularly those of poor and minority populations. The communities surrounding these centers have benefited from access to local researchers and resources to help them address the important environmental health issues in their homes, neighborhoods, and work-places. Olden then put forward the idea that community groups could apply for, and receive, funding from the NIEHS/NIH for research programs and outreach projects of their own. Such a grants program was a novel undertaking for a component of the NIH, which, as the nation’s largest biomedical research enterprise, traditionally funds only universities and research institutions.

Tradition notwithstanding, Olden initiated the Environmental Justice Grants Program and the Community-Based Prevention/Intervention Research Program. These programs have been the basis for science capacity building in local communities, such that several grantees have been able to compete successfully for much larger federal research programs in collaboration with neighboring academic institutions. Olden was one of the first NIH directors to initiate programs specifically designed to address health disparities, a measure that has now been adopted by the entire NIH. These programs are examples of many that Olden initiated with the goal of translating basic and clinical environmental health science into public health practice.

Central to Olden’s philosophy of translational research has been his belief that the research process must be a two-way street—that communication does not simply flow from the “ivory towers,” but that the people charged with setting national research agendas must be responsive to the needs and messages of the publics they serve. With this in mind, he invited all environmental health stakeholders to come to the table and voice their concerns. He made openness, transparency, and accessibility integral to the processes of the NTP. In 1998 he instituted a series of national town meetings in cities across the United States, where residents were invited to an open forum that he himself attended for discussion of the environmental health issues of importance to them. These discussions have become part of the basis for a national environmental health agenda. Olden has also taken bold steps, through the creation of the NIEHS Public Interest Liaison Group, to involve the public in brainstorming sessions with scientists, environmental professionals, and organizations concerned with diseases such as Parkinson disease, breast cancer, and respiratory disease, or that represent at-risk populations such as children, women, and minorities, to help the institute determine future research priorities.

One of the most visible aspects of Olden’s concerted effort to provide a forum for discussion of environmental health issues and communication of the most current scientific research is *EHP* itself. Almost immediately Olden recognized that the journal should work to become the most effective vehicle possible for accomplishing these goals. He began by directing the development of *EHP* into a monthly publication with an environmental news section written to be comprehensible to a lay audience. Over the years, *EHP* has expanded internally, adding sections on children’s health, environmental medicine, and toxicogenomics; the journal has also expanded internationally, publishing a Chinese-language edition and a dedicated section in the Spanish-language journal *Ciencia y Trabajo*. In a bold step forward for science communication, Olden deemed in 2003 that *EHP* would become entirely open access, and thus the information in it freely available to anyone in the world.

Through his vision, Olden has dramatically improved the visibility and reputation of the NIEHS while making its research programs more responsive to the needs of the American people. The institute’s emphasis on prevention and intervention is consistent with both public health and economic policies placing a high premium on disease prevention and the elimination of uncertainties in human risk assessment that lead to regulatory gridlock. Building on the core foundation of the NIEHS, Olden has created a host of programs to respond to new challenges and new opportunities. With his departure, Olden can rest safe in the knowledge that his leadership has ensured an NIEHS, and an *EHP*, that are poised to meet their futures as well.

## Figures and Tables

**Figure:**